# Supplementary material for: Estrogen Receptor β4 Regulates Chemotherapy Resistance and Induces Cancer Stem Cells in Triple Negative Breast Cancer
Source: Int J Mol Sci. 2023 Mar 20;24(6):5867. doi: 10.3390/ijms24065867 (PMC10058198; doi:10.3390/ijms24065867)
Supplement: Supplementary file 1 [file ijms-24-05867-s001.zip › ijms-2101694-supplementary.pdf]

International Journal of
Molecular Sciences

MDPI

Supplementary Material

Figure S1: MCF-10A pluripotent markers induced by ERβ4 expression

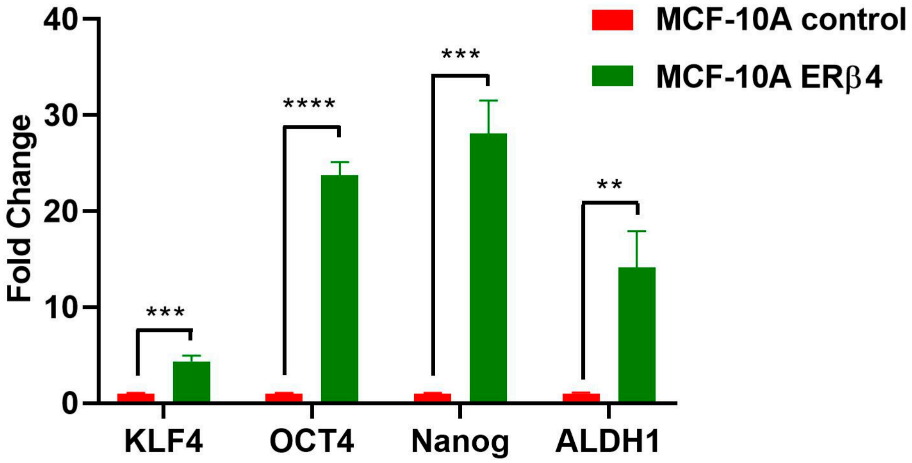

Figure S1. MCF-10A cells control transduced and transduced with Lenti- ERβ4. Pluripotent factor mRNA levels of KLF4, OCT4, NANOG and the stem cell marker ALDH1 estimated by quantitative PCR. Two – tailed Student's t-test was used ***P< 0.01, ***P < 0.001, ****P < 0.00001, ns non-significant.*

Figure S2: ERB4 knock down using CRSPR

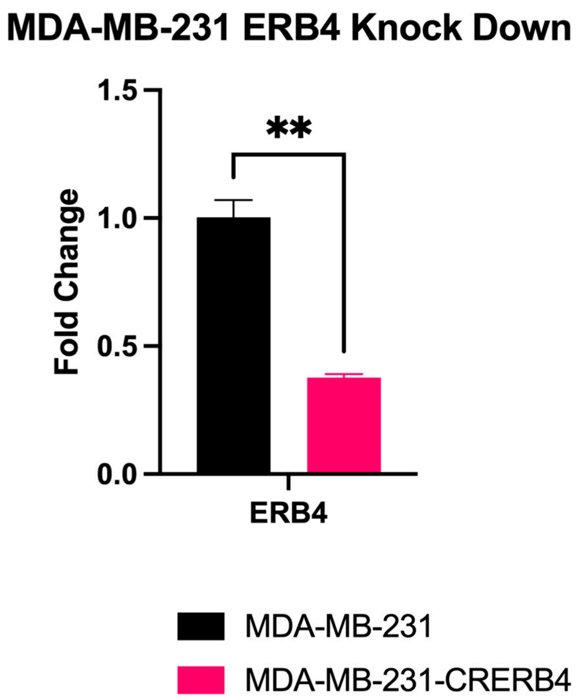

Figure S2. Expression level of ERβ4 mRNA in CRERB4 MDA-MB-231 cells compared to WT cells using qPCR. Two – tailed Student's t-test was used ***P< 0.01,*

Figure S3: *Knockdown of ERβ1 using DsiRNA*

A

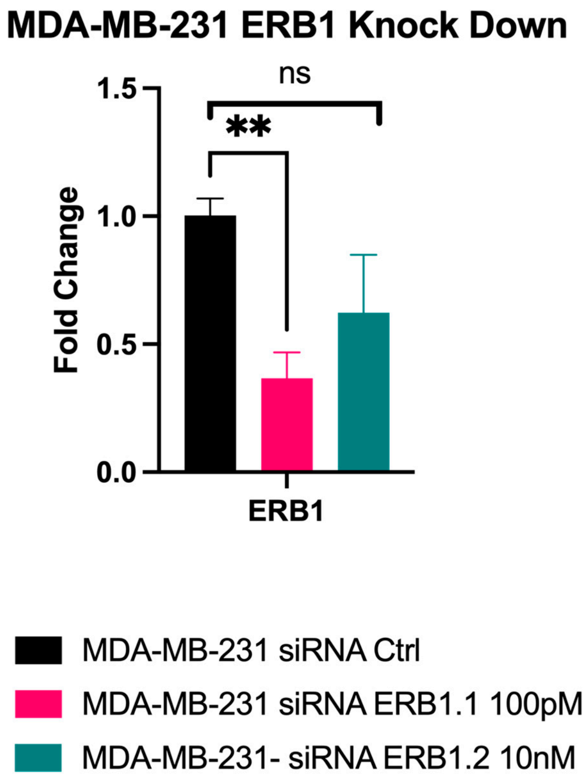

B

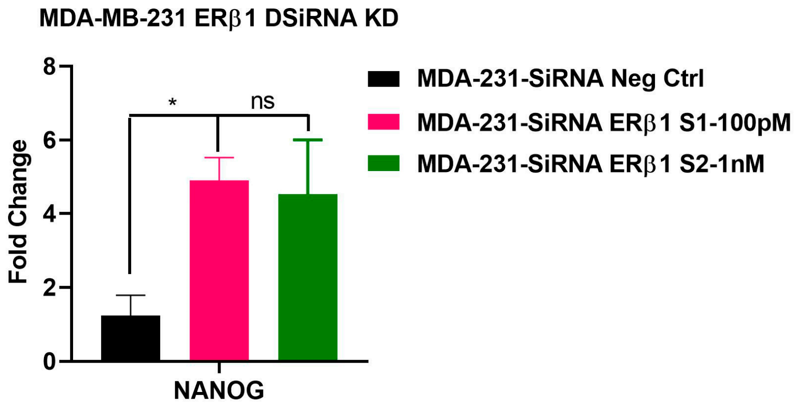

Figure S3. MDA-MB-231 cells transfected with two different DsiRNA at different concentrations A. knock down ERβ1. B. Increase of Nanog expression after ERβ1 knock down Two – tailed Student's t-test was used ***P< 0.01, ns non-significant.*

Figure S4: Count of SORE6+ cells in ERB4 overexpressing cells

A

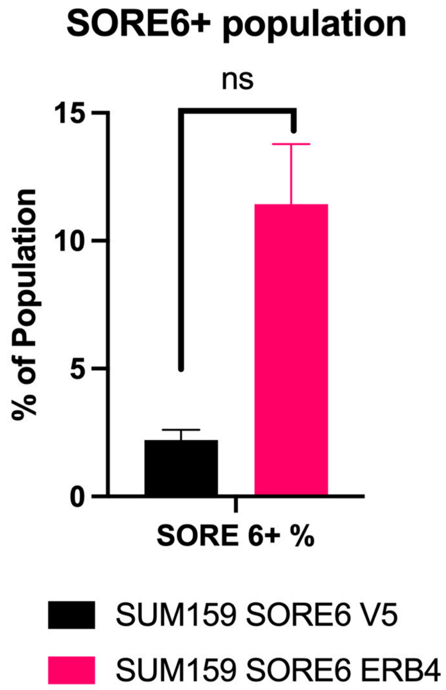

B

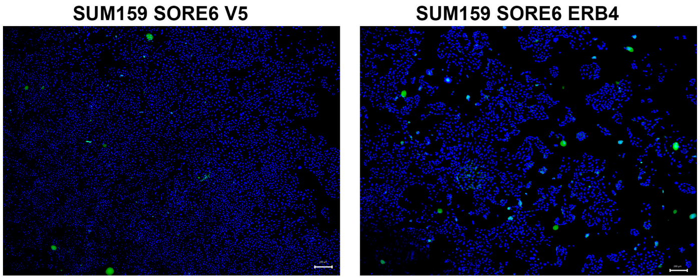

Figure S4. Stable SORE6 SUM159 cells were transfected with empty Lentivirus Vector, and vector expressing ERB4. The cells were grown in duplicate on glass slide and then imaged with Keyence BZ X-800. A. The SORE6-GFP+ cells were counted using the BZX-800 Analyzer and expressed as a ratio of all DAPI stained cells. Two – tailed Student's t-test was used *ns non-significant*. B. Images used for counting the scale bars is 200 µm.

Figure S5: Expression of ABCG2 in ERB4 overexpressing cells

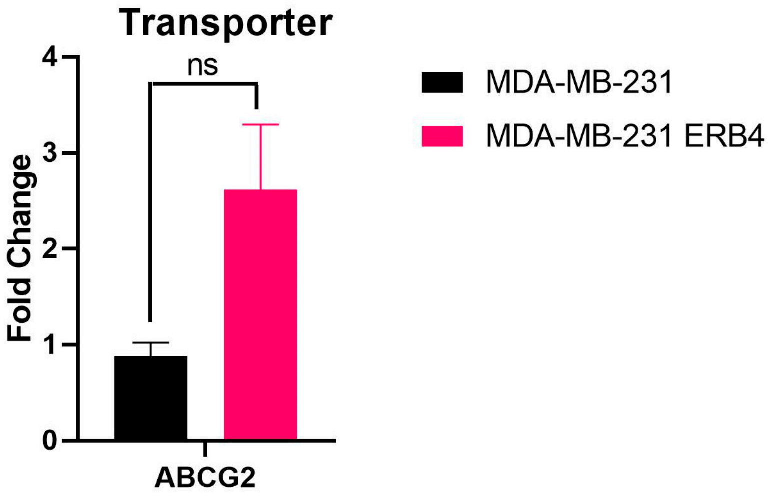

Figure S5. MDA-MB-231 transfected with empty Lentivirus Vector, and vector expressing ERB4. The cells were grown in 6 well plates. Expression of ABCG2 was performed using qPCR. Two – tailed Student's t-test was used *ns non-significant.*

Figure S6. Over expression of ERβ4 in SUM159 and MDA-MB-231 cells.

A

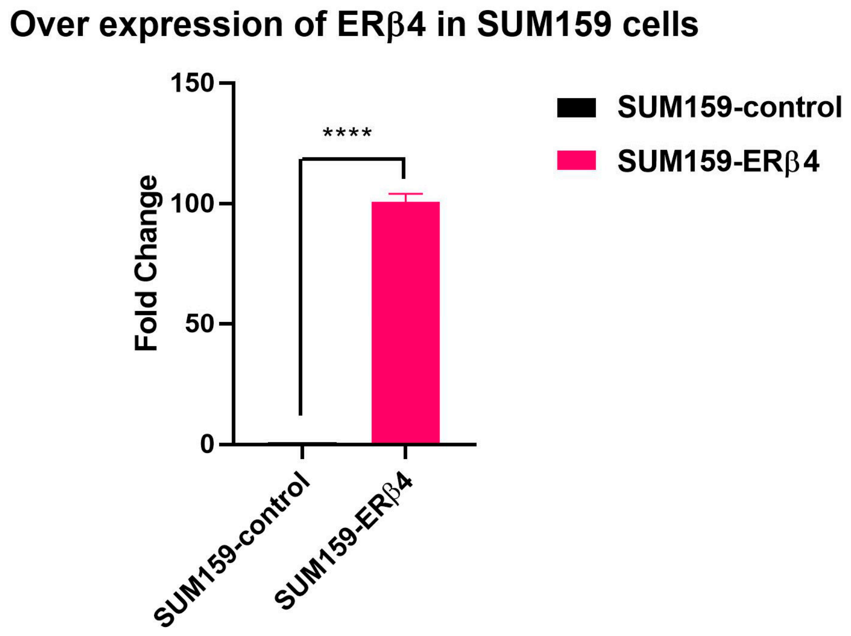

B

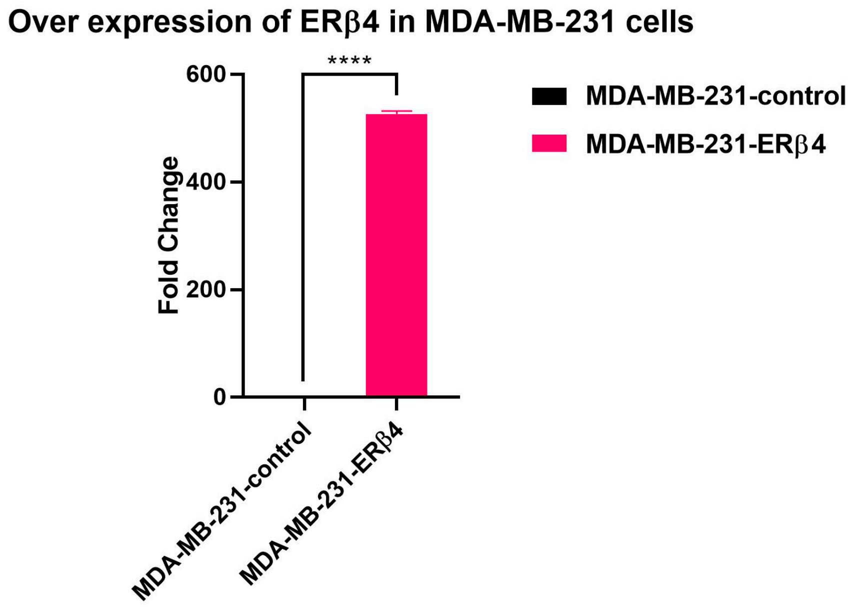

Figure S6. A. SUM159 and B. MDA-MB-231 transfected with empty Lentivirus Vector, and vector expressing ERβ4. The cells were grown in 6 well plates. Expression of ERβ4 was performed using qPCR. Two – tailed Student's t-test was used *ns non-significant.*
